# Management of a Chronic Skin Disease in Primary Care: An Analysis of Early-Career General Practitioners’ Consultations Involving Psoriasis

**DOI:** 10.5826/dpc.1103a55

**Published:** 2021-05-20

**Authors:** Sameerah Nawaz, Amanda Tapley, Andrew R. Davey, Mieke L. van Driel, Alison Fielding, Elizabeth G. Holliday, Jean Ball, Irena Patsan, Alyse Berrigan, Simon Morgan, Neil A. Spike, Kristen FitzGerald, Parker Magin

**Affiliations:** 1 GP Synergy, Regional Training Organisation, Liverpool, NSW, Australia; 2 The University of Newcastle, School of Medicine and Public Health, Callaghan, NSW, Australia; 3 GP Synergy, Regional Training Organisation, NSW & ACT Research and Evaluation Unit, Mayfield West, NSW, Australia; 4 The University of Queensland Faculty of Medicine, Primary Care Clinical Unit, Brisbane, QLD, Australia; 5 Hunter Medical Research Institute, Clinical Research Design, IT and Statistical Support Unit (CReDITSS), New Lambton, NSW, Australia; 6 Eastern Victoria GP Training, General Practice Training Organisation, Melbourne, Australia; 7 The University of Melbourne, Department of General Practice, Melbourne, VIC, Australia; 8 University of Tasmania, School of Medicine, Hobart, TAS, Australia; 9 General Practice Training Tasmania (GPTT), Regional Training Organisation, Hobart, TAS, Australia

**Keywords:** general practice, family practice, psoriasis, continuity of patient care, medical and graduate education, chronic disease

## Abstract

**Background:**

The management of psoriasis by general practitioners (GPs) is vital, given its prevalence, chronicity, and associated physical and psychosocial co-morbidities. However, there is little information on how GPs (including early-career GPs) manage psoriasis.

**Objectives:**

This study assessed the frequency with which Australian specialist GP vocational trainees (‘registrars’) provide psoriasis care and the associations of that clinical experience.

**Methods:**

A cross-sectional analysis was done of data from the ReCEnT study, an ongoing multi-site cohort study of Australian GP registrars’ experiences during vocational training. In ReCEnT, 60 consecutive consultations are recorded 3 times (6-monthly) during each registrar’s training. The outcome factor for this analysis was a problem/diagnosis being psoriasis, and independent variables were related to registrar, patient, practice and consultation factors. This study analysed 17 rounds of data collection (2010–2017) using univariate and multivariable regression.

**Results:**

Data from 1,741 registrars regarding 241,888 consultations and 377,980 problems/diagnoses were analysed. Psoriasis comprised 0.15% (n=550) of all problems/diagnoses (95% CI, 0.13–0.16). Significant patient multivariable associations of a problem/diagnosis being psoriasis included age, gender, being new to a practice or a registrar, and psoriasis being an existing problem rather than a new diagnosis. Significant registrar associations included seeking in-consultation information/assistance, not scheduling a follow-up appointment, prescribing medication, and generating learning goals.

**Conclusions:**

Australian registrars have modest training exposure to psoriasis and may find psoriasis management challenging. Furthermore, continuity of care (essential for optimal chronic disease management) was modest. The findings have implications for GPs’ approaches to the management of psoriasis more widely as well for general practice education and training policies.

## Introduction

Psoriasis is an autoimmune and autoinflammatory condition resulting in chronic inflammation of the skin and, typically, a prolonged clinical course. There are different phenotypes associated with varied genetics, immune pathophysiology and symptoms [1]. The most common type, chronic plaque psoriasis, comprises 90% of cases [2]. Other types of psoriasis include guttate psoriasis, generalised pustular psoriasis, palmoplantar psoriasis and erythrodermic psoriasis [3]. Psoriasis can also be classified into two types according to age at onset. Type I psoriasis is the more common type, comprising 75% of all cases, and is associated with early onset (before age 40) and more severe disease (HLA-Cw*0602 positive.) Type II psoriasis is HLA-Cw*0602 negative, and occurs later in life [4].

There is a wide variation in the prevalence of psoriasis among different populations. Variations in prevalence may be attributable to study design (eg population-based versus hospital based, or self-reported versus clinician-diagnosed) or to geographic and environmental factors [5]. It is clear that Australia has a particularly high prevalence of psoriasis [6]. Psoriasis in Australian Aboriginal people, however, is rare or absent [7].

The burden of psoriasis on individuals can include significant physical, social and psychological impacts [8,9]. Psoriasis is associated with lifestyle factors including obesity, smoking and alcohol use [10]. Independent of these risk factors, psoriasis is associated with multiple medical comorbidities including psoriatic arthritis (30%), inflammatory bowel disease, metabolic syndrome, non-alcoholic fatty liver disease, cardiovascular disease, myocardial infarction, diabetes, chronic renal disease and stroke [11–14]. Psychiatric and psychosocial comorbidities and impairment of quality of life are common and problematic [9,15,16]. Additionally, treatments for psoriasis can have adverse effects involving multiple systems [8]. Thus, given the prevalence, chronicity, and physical and psychosocial comorbidities of psoriasis, it is vital that general practitioners (GPs) are continually engaged in – and have expertise in – the management of psoriasis.

Australian GPs (family physicians) encounter psoriasis with a frequency of approximately 0.2 in every 100 patient visits [17]. Patients’ experiences of GP consultations for psoriasis may often be negative, with many finding that GPs have limited knowledge and, more importantly, limited appreciation of its impact on patients’ lives [18].

Registrars are early-career GPs practising within a national specialist general practice training program. They are establishing what may be long-lasting practice patterns in a clinical context of having been underprepared for managing skin disease by their undergraduate and junior doctor experiences [19–21]. An understanding of consultation and management patterns, as with skin disease in general, can lead to a better understanding and improvement of psoriasis management in the community setting [22].

In this study, we aimed to establish the frequency and associations of psoriasis consultations in registrars’ clinical experience. In view of the chronicity of psoriasis, we were particularly interested in the associations of factors related to the continuity of care.

## Materials and Methods

This was a cross-sectional analysis of data from the Registrar Clinical Encounters in Training (ReCEnT) study.

### ReCEnT

ReCEnT is an ongoing multi-site cohort study of Australian GP registrars. Participants are registrars in the 3 compulsory general-practice-based training terms of Australian GP vocational training. Registrars practice within an apprenticeship-like training model [23], but with considerable clinical autonomy (whereby they can request assistance from their experienced GP supervisor, if required). From 2010 to 2015, 5 of Australia’s 17 Regional Training Providers (RTPs) participated and, since 2016, 3 of Australia’s 9 Regional Training Organisations (RTOs) have participated (in 2016 there was a major restructuring of Australian GP training). RTOs and RTPs are geographically defined, not-for-profit educational organisations. Hereafter, RTOs, RTPs and RTO subregions are referred to as ‘regions’. At the time of this analysis, individual regions had contributed from 3 to 17 rounds of data collection.

The methodology has been described elsewhere [24]. Briefly, registrars undertake data collection once per 6-month training term as an integral component of their educational program [25]. The data are used to compose detailed written feedback reports for registrar reflection on their clinical practice and educational and training needs. Registrars may provide written informed consent for their de-identified data to also be used for research purposes. Initial data collection involves registrar demographic, educational, and work experience, plus characteristics of the practice in which they are currently working.

Approximately mid-way through the training term, registrars record the details of 60 consecutive patient consultations on a paper-based Case Report Form. As data collection is designed to reflect a ‘normal’ week of general practice, consultations in a specialised clinic, eg vaccination clinic, are excluded. Only office-based consultations (not home visits or nursing home visits) are recorded.

### Outcome Factor

The outcome factor in this study was a problem/diagnosis being ‘psoriasis’. ‘Psoriasis’ was defined as the ICPC-2 code S91 001 (Psoriasis). In ReCEnT, problems/diagnoses are coded according to the International Classification of Primary Care, second edition classification system (ICPC-2 plus) [26].

## Independent Variables

Independent variables listed in Table 2 related to registrar, patient, practice, and consultation (including in-consultation educational factors). Each practice’s postal code was used to define the degree of rurality, according to the Australian Standard Geographical Classification-Remoteness Area (ASGC-RA) classification [27] and the location’s Socioeconomic Index for Area (SEIFA) Relative Index of Disadvantage [28].

### Statistical Analysis

Analysis was performed on 17 rounds of data collected from 2010 to 2017. Analysis was at the level of individual problem/diagnosis. The proportions of registrars’ problems/diagnoses that were psoriasis and of consultations involving a psoriasis problem/diagnosis were calculated, with 95% confidence intervals, adjusted for clustering within registrars. To test associations of a problem/diagnosis being psoriasis, simple and multiple logistic regression were used within the generalised estimating equations (GEE) framework to account for clustering of patients within registrars. (This is the lowest level of clustering in the study design: previous analyses of the ReCEnT data set have demonstrated that also adjusting for clustering at practice level is non-contributory.) All variables with a P value less than .20 in the univariate analysis were included in the multiple regression model. Covariates with P >.2 in the resulting multivariable model were tested for removal. If the covariate’s removal did not substantively change the model, the covariate was removed from the final model.

The outcome proportion for psoriasis was very low (0.15%), which can cause problems of complete or quasi-complete separation of data. This means that the outcome variable separates a predictor variable or a combination of predictor variables completely. To check for the impact of data separation, logistic regression coefficients were estimated using Firth’s penalised likelihood in a sensitivity analysis. Coefficients differed negligibly in the sensitivity analysis, and thus results from standard logistic regression were reported. Results from the logistic regression were reported as odds ratios (OR) with 95% confidence intervals.

In order to examine 3 separate issues within our overall research question, 3 models were built, each with ‘psoriasis being the problem/diagnosis’ as the dependent variable:

- To examine the question of associations of a problem/diagnosis being psoriasis, patient, practice, and registrar independent variables were entered in an initial regression model.
- To examine the question of in-consultation differences of a psoriasis problem/diagnosis problem compared with other problems/diagnoses, the above variables were entered in a model along with the following additional variables: consultation duration, information/assistance accessed by the registrar, number of problems/diagnoses dealt with in the consultation, and consultation duration.
- To examine the question of whether actions arising from managing psoriasis differ from those arising from other problems/diagnoses, all variables entered in the previous 2 models were entered in a new model along with the following additional variables: learning goals generated by the registrar, follow-up organised, specialist referrals made, and pathology tests and imaging exams ordered.

The rationale for the building of the 3 models was that whether a patient presents with psoriasis (our first question) will plausibly be influenced by patient, registrar, and practice factors, but evaluation of this question may be biased by inclusion in the model of factors operating once the consultation is progressing. Similarly, evaluation of the content of the consultation (our second question), may be biased by the inclusion in the model of actions arising from the consultation.

The frequencies with which medicines were prescribed for psoriasis, and with which referrals were made, were calculated. The frequency with which sources of in-consultation information or assistance were sought was also calculated.

Statistical analyses were programmed using STATA 14.0 and SAS v9.4. P values <.05 were considered statistically significant.

### Ethics Approval

The ReCEnT project has approval from the University of Newcastle Human Research Ethics Committee (Reference H-2009-0323).

## Results

The study analysed data from a total of 1,741 registrars (response rate, 96.0%). The demographics of participating registrars are presented in Table 1. The registrars provided data on 241,888 consultations and 377,980 problems/diagnoses. Of all problems/diagnoses, 550 (0.15% [95% CI, 0.13–0.16]) were a psoriasis problem/diagnosis. This equates to 0.22% of all consultations (95% CI, 0.20–0.24).

### Associations of Psoriasis Problems/Diagnoses Managed by Registrars

Table 2 presents the characteristics associated with a problem/diagnosis being psoriasis in the 1,741 registrars’ practices.

Table 3 presents the results of simple and multiple logistic regression analyses. Significant patient associations on multivariable analysis of the problem/diagnosis being psoriasis were age group 15–34 (compared to younger or older patients), male gender (OR = 0.7 for female gender), the patient being new to the practice (OR = 2.6 compared to an existing patient of the practice), the patient being new to the registrar (OR = 2.1 compared to an existing patient of the practice), and the problem being an existing one (OR = 0.1 for a new problem). The significant registrar association was training term 3 (OR = 1.3 compared to term 1). The significant multivariable consultation association of the problem/diagnosis being psoriasis was seeking in-consultation information or assistance (OR = 2.4).

Significant multivariable associations with factors arising from the registrar’s management of the problem/diagnosis included follow-up being less likely (OR = 0.7 for organising follow-up), medication being prescribed (OR = 3.8), and learning goals being generated (OR = 2.8).

Medication was prescribed for 435 (79%) of psoriasis problems/diagnoses. The most common medicines prescribed for psoriasis were betamethasone (31% of all prescriptions for psoriasis), mometasone (21%) and calcipotriol and combinations (20.0%). See Table 4 for the list of most commonly prescribed medications.

Only 180 patients with psoriasis problems/diagnoses (34%) had seen the registrar previously (for any reason), and for only 199 psoriasis problems/diagnoses (36%) was follow-up organised. In 89% of instances, this was with the registrar personally. When seen for psoriasis, 13% of patients were referred to a dermatologist, with 9% of new diagnoses being referred and 15% of previously diagnosed patients being referred.

In-consultation information or assistance was accessed for 29% of psoriasis problems/diagnoses. The sources of in-consultation assistance or information used were electronic sources (64%), the registrar’s supervisor (33% of instances), hard-copy sources (6%), and specialists (4%).

## Discussion

### Frequency of Psoriasis Problems/Diagnoses Encountered

We found that 0.22% of registrar consultations involved a psoriasis diagnosis/problem. While this is comparable to the frequency with which established GPs encounter psoriasis [17], this equates to approximately once every 8 weeks, or approximately 10 times during registrars’ 3 core general practice training terms. This relative low frequency of registrars managing psoriasis (together with a lack of undergraduate and hospital doctor experience of skin disease) [19] suggests these early-career GPs may have unmet learning needs related to psoriasis. This is consistent with the association we found with high levels of seeking in-consultation information or assistance and generation of learning goals in relation to psoriasis compared to other problems.

### Patient Demographics Associated with Psoriasis

Our study showed that a patient being between the ages of 15 and 64 years was significantly associated with the problem/diagnosis being psoriasis (compared to younger or older patients). This is consistent with a previous study that found low rates of psoriasis in the paediatric population, with a peak starting at age 20 and declining after 60 [29]. Although previous studies have not found any consistent difference in prevalence by gender [6], our study showed a lower likelihood of registrars seeing psoriasis in females. A possible explanation is that psoriasis has been found to be more severe in males than in females, and our finding may reflect healthcare-seeking behaviours rather than community prevalence [30].

### Associations of a Psoriasis Problem/Diagnosis

The registrar being in term 3 (vs. term 1) of their training was significantly associated with a psoriasis problem/diagnosis. This, again, may reflect the paucity of dermatological experience and expertise of registrars entering general practice and increasing confidence and comfort in addressing psoriasis with more experience.

A large number of medications was prescribed by registrars for psoriasis. Most were (appropriately) topical steroids and vitamin D analogues. This (together with the strong association of psoriasis problems/diagnoses with in-consultation assistance-seeking) suggests that, despite their lack of experience in this area, consultation with information sources and the GP registrars’ supervisor may facilitate appropriate treatment.

### Implications for the Management of Psoriasis as a Chronic Disease

This study found a strong association of consultations for psoriasis being for existing psoriasis. However, there were also strong associations of the patient being new to the practice, new to the registrar, and less follow-up appointments being organised. This suggests that these patients may have inconsistent provision of primary care for this problem. That is, there is low continuity of care (to some extent informational and management continuity but, especially, low interpersonal continuity) [31, 32]. This is contrary to findings of patients with chronic disease in general within this study population, where chronic disease is associated with greater continuity of care [33]. This is of considerable importance as psoriasis is a chronic disease with much associated morbidity (both psychosocial co-morbidity and high levels of co-morbid non-dermatological physical disease) [8, 9, 11, 15, 16]. One explanation for our finding is that psoriasis is being managed primarily by specialist dermatologists. Even if this were the case, shared care with a generalist is essential given the physical and psychosocial co-morbidities of psoriasis. Optimal generalist care, as for all chronic conditions, should include continuity of care.

Another interpretation is that the patients lack continuity of care with any clinician and that they do not receive coordinated care appropriate to their condition. This may be influenced by patients often not valuing GPs’ expertise in skin disease (including psoriasis). A feeling that GPs do not appreciate patients’ experience of skin disease has been reported [18]. Further associations in our study may support this interpretation. We found a strong association with the prescription of medicines and specialist referral. An interpretation may be that many patients receive episodic care, often prompted by the need to renew psoriasis prescriptions and referrals,. They may be seeking a convenient prescriber performing a routine, discrete administrative task rather than a GP involved in ongoing, considered management of their chronic disease. Possible sequelae of such a situation are different providers delivering uncoordinated medicine regimens that do not facilitate a logical progression of treatment based on response to past medications [34–36]. Furthermore, holistic care of patients is likely to be compromised with a lack of addressing physical and psychosocial morbidities. Lack of continuity of care will also compromise shared decision-making which has been advocated in the management of skin conditions in general [22] and in psoriasis management [37] in particular.

In this context, our finding that when registrars do organise a follow-up appointment for psoriasis, it is usually with themselves (rather than another GP in the practice) should be noted. It may suggest that they appreciate the importance of, and are attempting to facilitate, interpersonal continuity of care for psoriasis.

### Implications for Education and Training

As we found evidence suggesting that registrars see little psoriasis in practice over the course of GP vocational training, targeted education and training in this area may be required. This could be addressed through structural changes within practice, appointment scheduling and policy regarding follow-up, along with concurrent patient education regarding the needs for continuity of care for management of their psoriasis, including shared decision-making and self-management [22].

## Conclusions

We have identified educational and organisational aspects of registrar psoriasis management that could be optimised with changes at a registrar, practice and patient level. Registrars see relatively little psoriasis in practice during vocational training, and evidence was found to suggest that registrars are finding psoriasis care challenging. This suggests opportunity for more education and training in this area. We also found evidence that psoriasis (a chronic disease with frequent comorbidity), despite registrars’ attempts, did not show the same pattern of continuity of care that is usually seen in optimal chronic disease management.

## Figures and Tables

**Table 1:** Participating trainee, trainee-term and practice characteristics

Variable	Class	n (%) or Mean (SD)
Registrar variables (n=1741)		
Registrar Gender	Female	1,116 (64.1)
Pathway registrar enrolled in (General or Rural)	Rural	446 (25.8)
International Medical Graduate or Qualified as a doctor in Australia	International Graduate	302 (17.5)
Registrar-term or practice-term variables (n=4072)		
Registrar Training Term	Term 1	1,613 (39.6)
	Term 2	1,471 (36.1)
	Term 3	988 (24.3)
Registrar age (years)	Mean (SD)	32.4 (6.1)
Registrar works full time	Yes	3079 (77.7)
Registrar worked at the current practice previously	Yes	996 (24.8)
Does the practice routinely bulk bill^1^ all patients	Yes	898 (22.4)
Number of GPs working at the practice	1–5	1,430 (36.2)
	6–9	2,526 (63.9)
Rurality of practice	Major City	2,443 (60.2)
	Inner Regional	1,021 (25.2)
	Outer regional, remote or very remote	594 (14.6)
SEIFA^2^ Index of practice	Mean (SD)	5.5 (2.8)

1 Bulk bill, to charge the entire costs of an episode of patient care to the Australian national health care system.

2 SEIFA = Socioeconomic Index for Area Relative Index of Disadvantage.

**Table 2:** Characteristics Associated With a Problem/Diagnosis in Registrars’ Practice Being Psoriasis

Variable	Class	Problem/Diagnosis, n (%)^1^		P
		Other	Psoriasis	
**Total**		377,430 (100)	550 (100)	-
**Patient Variables**				
Age group (years)	0–14	52,353 (14)	19 (4)	<.001
	15–34	98,406 (26)	149 (28)	
	35–64	147,691 (40)	278 (51)	
	≥65	73,227 (20)	95 (18)	
Gender	Male	138,926 (38)	251 (47)	<.001
	Female	229,392 (62)	288 (53)	
Aboriginal Torres Strait Islander status	No	348,082 (98)	501 (98)	.32
	Yes	5,657 (2)	11 (2)	
Non-English-speaking background	No	328,363 (92)	480 (94)	.23
	Yes	28,129 (8)	33 (6)	
Patient/practice status	Existing patient	158,290 (43)	180 (34)	<.001
	New to registrar	184,591 (50)	312 (58)	
	New to practice	25,953 (7)	43 (8)	
**Registrar Variables**				
Gender	Male	131,335 (35)	199 (36)	.54
	Female	246,095 (65)	351 (64)	
Works full time	No	84,158 (23)	117 (22)	.53
	Yes	283,552 (77)	422 (78)	
Term	Term 1	152,328 (40)	223 (41)	.14
	Term 2	134,357 (36)	177 (32)	
	Term 3	90,745 (24)	150 (27)	
Worked at practice previously	No	279,145 (75)	416 (76)	.42
	Yes	93,406 (25)	128 (24)	
Qualified as doctor in Australia	No	63,796 (17)	75 (14)	.058
	Yes	311,330 (83)	470 (86)	
Age (years)	Mean (SD)	32 (6)	32 (6)	.76
**Practice Variables**				
Practice size	Small	134,317 (37)	192 (35)	.58
	Large	232,596 (63)	349 (65)	
Routine bulk billing 2	No	289,580 (78)	418 (77)	.68
	Yes	83,118 (22)	125 (23)	
Rurality	Major city	225,666 (60)	337 (62)	.48
	Inner regions	94,719 (25)	125 (23)	
	Outer regions or remote	55,786 (15)	85 (16)	
Sub-region	1	100,799 (27)	146 (27)	.89
	2	36,149 (10)	46 (8)	
	3	49,566 (13)	74 (13)	
	4	151,255 (40)	227 (41)	
	5	10,400 (3)	12 (2)	
	6	29,261 (8)	45 (8)	
SEIFA (decile)	Mean (SD)	5 (3)	6 (3)	.28
**Consultation Variables**				
New problem seen	No	152,857 (44)	417 (84)	<.001
	Yes	192,886 (56)	80 (16)	
Sought help from any source	No	315,534 (84)	391 (71)	<.001
	Yes	61,896 (16)	159 (29)	
Consultation duration (minutes)	Mean (SD)	19 (10)	19 (10)	.47
Number of problems dealt with in the consultation	Mean (SD)	2 (1)	2 (1)	.002
**Consultation Outcome Variables**				
Imaging exam ordered	No	348,644 (92)	546 (99.3)	<.001
	Yes	28,786 (8)	4 (0.7)	
Pathology exam ordered	No	308,712 (82)	506 (92)	<.001
	Yes	68,718 (18)	44 (8)	
Learning goals generated	No	298,600 (83)	338 (65)	<.001
	Yes	61,465 (17)	179 (35)	
Follow-up ordered	No	211,640 (56)	351 (64)	<.001
	Yes	165,790 (44)	199 (36)	
Referral ordered	No	331,078 (88)	470 (85)	.11
	Yes	46,352 (12)	80 (15)	
Medication prescribed	No	211,612 (56)	115 (21)	<.001
	Yes	165,818 (44)	435 (79)	

SEIFA = Socioeconomic Index for Area Relative Index of Disadvantage.

1 Values are n (%) unless otherwise indicated. Percentages are out of the total number of problems/diagnoses.

2 Bulk bill, to charge the entire costs of an episode of patient care to the Australian national health care system.

**Table 3:** Associations of a Problem/Diagnosis in the Registrars’ Practice Being Psoriasis: Univariate and Multivariable Models

Variable	Class	Univariate		Adjusted	
		OR (95% CI)	P	OR (95% CI)	P
***Patient, Registrar and Practice Variables***					
Patient Age group (Referent: 15–34 years)	0–14	0.2 (0.2, 0.4)	<.001	0.3 (0.2, 0.5)	<.001
	35–64	1.2 (1.0, 1.5)	.041	1.0 (0.8, 1.3)	.82
	≥65	0.9 (0.7, 1.1)	.24	0.7 (0.5, 0.9)	.004
Patient Gender	Female	0.7 (0.6, 0.8)	<.001	0.7 (0.6, 0.8)	<.001
Patient/practice status (Referent: existing patient)	New to practice	1.5 (1.1, 2.0)	.023	2.6 (1.8, 3.7)	<.001
	New to registrar	1.5 (1.2, 1.8)	<.001	2.1 (1.7, 2.5)	<.001
Term (Referent: term 1)	Term 2	0.9 (0.7, 1.1)	.31	1.0 (0.8, 1.2)	.75
	Term 3	1.1 (0.9, 1.4)	.27	1.3 (1.0, 1.6)	.045
New problem seen	Yes	0.2 (0.1, 0.2)	<.001	0.1 (0.1, 0.2)	<.001
***Consultation Variables (adjusted for above variables)***					
Sought help from any source	Yes	2.1 (1.7, 2.5)	<.0001	2.4 (2.0, 3.0)	<.001
***Consultation Outcome Variables (adjusted for above variables)***					
Imaging ordered	Yes	0.1 (0.0, 0.2)	<.001	0.1 (0.0, 0.4)	<.001
Follow-up ordered	Yes	0.7 (0.6, 0.9)	<.001	0.7 (0.6, 0.8)	<.001
Learning goals generated	Yes	2.6 (2.2, 3.1)	<.001	2.8 (2.1, 3.5)	<.001
Pathology ordered	Yes	0.4 (0.3, 0.5)	<.001	0.4 (0.3, 0.6)	<.001
Medication prescribed	Yes	4.8 (3.9, 6.0)	<.001	3.8 (3.0, 4.8)	<.001

**Table 4:** Most Commonly Prescribed Medicines for Psoriasis

Medication	Prescriptions, n (%)	Prescriptions for Psoriasis, n (%)	New Prescriptions, n (%)
Betamethasone	193	31.4	35.8
Mometasone	129	21.0	35.7
Calcipotriol and combinations	123	20.0	33.3
Methylprednisolone aceponate	35	5.7	80.0
Hydrocortisone	27	4.4	48.1
Methotrexate	13	2.1	7.7
Salicylic acid	13	2.1	69.2
Triamcinolone	12	2.0	41.7
Methylprednisolone	11	1.8	63.6
Clobetasol	6	1.0	33.3
Prednisolone	6	1.0	50.0
Dithranol and combinations	5	0.8	80.0
